# Real-world Treatment Sequencing in Patients with Metastatic Castration-resistant Prostate Cancer: Results from the Prospective, International, Observational Prostate Cancer Registry

**DOI:** 10.1016/j.euros.2022.08.018

**Published:** 2022-09-17

**Authors:** Anders Bjartell, Luis Costa, Gero Kramer, Bogdan Zurawski, Luca Galli, Patrick Werbrouck, Thorsten Ecke, Omi Parikh, Mostefa Bennamoun, Camilo Garcia Freire, Avivit Peer, Börje Ljungberg, Irfan Cicin, Emma Smith, Martin Lukac, Robert Wapenaar, Simon Chowdhury

**Affiliations:** a Department of Urology, Skåne University Hospital, Lund University, Malmö, Sweden; b Oncology Division, Centro Hospitalar Universitário Lisboa Norte, Lisbon, Portugal; c Faculdade de Medicina da Universidade de Lisboa, Lisbon, Portugal; d Instituto de Medicina Molecular João Lobo Antunes, Lisbon, Portugal; e Department of Urology, Medical University of Vienna, Vienna, Austria; f Centrum Onkologii im. Prof. F. Lukaszczyka w Bydgoszczy, Dzial Onkologii Klinicznej, Ambulatorium Chemioterapii, Bydgoszcz, Poland; g Pisana University Hospital, Pisa, Italy; h AZ Groeninge, Kortrijk, Belgium; i Helios Klinikum Bad Saarow, Bad Saarow, Germany; j Royal Blackburn Hospital, Blackburn, UK; k Institut Mutualiste Montsouris, Paris, France; l Hospital Clínico Universitario de Santiago, Servicio de Urología, Santiago de Compostela, Spain; m Rambam Medical Center, Haifa, Israel; n Department of Surgical and Perioperative Sciences, Urology and Andrology, Umeå University, Umeå, Sweden; o Trakya University Hospital Medical Oncology Department, Edirne, Turkey; p Janssen-Cilag, High Wycombe, UK; q Parexel International Czech Republic sro, on behalf of Janssen Pharmaceutica NV, Beerse, Belgium; r Janssen-Cilag BV, Breda, The Netherlands; s Guy’s and St Thomas’ NHS Foundation Trust and Sarah Cannon Research Institute, London, UK

**Keywords:** Abiraterone acetate, Androgen deprivation therapy, Antiandrogen, Enzalutamide, Cabazitaxel, Castration-resistant prostate cancer, Docetaxel, Ra-223, Real-world evidence

## Abstract

**Background:**

Prostate cancer has a multifaceted treatment pattern. Evidence is lacking for optimal treatment sequences for metastatic castration-resistant prostate cancer (mCRPC).

**Objective:**

To increase the understanding of real-world treatment pathways and outcomes in patients with mCRPC.

**Design, setting, and participants:**

A prospective, noninterventional, real-world analysis of 3003 patients with mCRPC in the Prostate Cancer Registry (PCR; NCT02236637) from June 14, 2013 to July 9, 2018 was conducted.

**Intervention:**

Patients received first- and second-line hormonal treatment and chemotherapy as follows: abiraterone acetate plus prednisone (abiraterone)-docetaxel (ABI-DOCE), abiraterone-enzalutamide (ABI-ENZA), abiraterone–radium-223 (ABI-RAD), docetaxel-abiraterone (DOCE-ABI), docetaxel-cabazitaxel (DOCE-CABA), docetaxel-enzalutamide (DOCE-ENZA), and enzalutamide-docetaxel (ENZA-DOCE).

**Outcome measurements and statistical analysis:**

Baseline patient characteristics, quality of life, mCRPC treatments, and efficacy outcomes (progression and survival) were presented descriptively.

**Results and limitations:**

Data from 727 patients were eligible for the analysis (ABI-DOCE *n* = 178, ABI-ENZA *n* = 99, ABI-RAD *n* = 27, DOCE-ABI *n* = 191, DOCE-CABA *n* = 74, DOCE-ENZA *n* = 116, and ENZA-DOCE *n* = 42). Demographics and disease characteristics among patients between different sequences varied greatly. Most patients who started on abiraterone or enzalutamide stopped therapy because of disease progression. No randomisation to allow treatment/sequence comparisons limited this observational study.

**Conclusions:**

The real-world PCR data complement clinical trial data, reflecting more highly selected patient populations than seen in routine clinical practice. Baseline characteristics play a role in mCRPC first-line treatment selection, but other factors, such as treatment availability, have an impact. Efficacy observations are limited and should be interpreted with caution.

**Patient summary:**

Baseline characteristics appear to have a role in the first-line treatment selection of metastatic castration-resistant prostate cancer in the real-world setting. First-line abiraterone acetate plus prednisone seems to be the preferred treatment option for older patients and those with lower Gleason scores, first-line docetaxel for younger patients and those with more advanced disease, and first-line enzalutamide for patients with fewer metastases and more favourable performance status. The benefit to patients from these observations remains unknown.

## Take Home Message

Patient characteristics appear to influence the first-line treatment of metastatic castration-resistant prostate cancer. Patients older and with lower Gleason scores tended to receive abiraterone, those younger and with more advanced disease tended to receive docetaxel, and those with fewer metastases and more favourable performance tended to receive enzalutamide. The benefit to patients from these observations remains unknown.

## 1 Introduction

Prostate cancer is the second most frequently occurring cancer and the fifth leading cause of cancer death in men worldwide [1], with an estimated 1.41 million new cases globally in 2020 [2]. Since 2010, several new agents have been approved for metastatic castration-resistant prostate cancer (mCRPC) that have demonstrated an overall survival (OS) benefit in clinical studies. However, robust evidence based on randomised trials for optimal treatment sequences for mCRPC is lacking [3], [4], [5], with no high-level recommendations in guidelines or expert consensus [6].

Current prostate cancer registries primarily report incidence and mortality data in selected countries and regions [7], [8]. They are not designed to enable comprehensive assessment of patient characteristics that can determine optimal treatment allocation, necessitating further registry studies. The Prostate Cancer Registry (PCR; NCT02236637) was initiated to document the characteristics and disease management of patients with mCRPC in routine clinical practice, independent of the treatment used. With >3000 enrolled patients, the PCR is the largest international registry of patients with mCRPC to date, with participation from 199 centres experienced in treating prostate cancer in 16 countries (Austria, Belgium, France, Germany, Israel, Italy, Luxembourg, Poland, Portugal, Russia, Slovenia, Spain, Sweden, Switzerland, Turkey, and the UK). The PCR was designed specifically to record the initiation of a new systemic mCRPC treatment during surveillance within routine clinical practice. Data from the PCR have shown the effectiveness, in parallel, of first-line abiraterone acetate plus prednisone (referred to as abiraterone hereafter), enzalutamide, and docetaxel, including in patients with comorbidities, such as cardiovascular disease or diabetes mellitus, and in patients with visceral metastases [9]. An analysis of PCR data has also recently shown the efficacy and tolerability of abiraterone as first- and second-line treatment for mCRPC in the real-world setting [10].

This analysis of data from the PCR was conducted to increase the understanding of real-world treatment pathways and outcomes in a broad group of patients treated with abiraterone, enzalutamide, or docetaxel as their initial treatment for mCRPC. It uses real-world information on baseline demographics and disease characteristics of patients with mCRPC who received sequential treatment with hormonal therapy and chemotherapy in this setting.

## 2 Patients and methods

This was a prospective, noninterventional, multicentre PCR data analysis of men with mCRPC, from June 14, 2013 to July 9, 2018; data from the PCR have been described previously [9]. This analysis was limited to men included in the PCR treated with abiraterone, enzalutamide, or docetaxel as first-line mCRPC therapy who had not received previous systemic mCRPC treatment. Abiraterone and enzalutamide were not routinely available for the treatment of mCRPC in all 16 countries at the start of the registry.

The study timeline is shown in Supplementary Figure 1. For each patient, first-line and subsequent mCRPC treatments were recorded until the PCR ended (for up to 3 yr). The criteria for inclusion of sequences were as follows: (1) first- and second-line treatments were to be used as single agents in addition to androgen deprivation therapy and approved by the European Medicines Agency for use in this patient population (ie, no off-label use) and (2) patient numbers in the sequence were to be adequate for a reliable analysis (≥25 patients with a sequence involving two lines of treatment in mCRPC).

Observational data collected for each treatment sequence included baseline patient demographics; disease and health-related quality of life characteristics at baseline and the start of second-line treatment, and their changes from baseline; reasons for starting and stopping mCRPC treatment; and efficacy outcomes.

Disease and health-related quality of life data included Eastern Cooperative Oncology Group (ECOG) performance status; prostate-specific antigen (PSA), lactate dehydrogenase, alkaline phosphatase, and haemoglobin levels; and EuroQol 5-dimension 5-level questionnaire (EQ-5D-5L) pain/discomfort dimension. Efficacy outcomes were time to progression (TTP), measured from the start of first treatment to the start of second treatment, progression-free survival 2 (PFS2), and OS. Definitions used for reporting efficacy outcomes are provided in the Supplementary material.

Treatment decisions were made at the discretion of the treating physician, as per routine clinical practice, and only data available from clinical practice were collected. The treating physician could be either an oncologist or a urologist, designation of which was not recorded in the registry. Prior disease history and management data were collected at study inclusion. Clinical data were collected at study inclusion and prospectively every 3 mo during routine follow-up (at least every 3 mo per protocol) over the 3-yr study period, with clinical trial levels of monitoring quality.

The study was conducted according to the applicable regulatory prerequisites. Prior to data collection, all patients provided informed consent in accordance with local requirements. The study was approved by the ethics committees of every country that participated and was conducted in accordance with the Declaration of Helsinki.

### 2.1 Statistical methods

Descriptive statistics were used to present observed data from routine clinical practice by treatment sequence for three first-line treatment groups. Percent data for outcomes were calculated from the number evaluable for each parameter as the denominator. Kaplan-Meier methods were used to analyse time-to-event variables (TTP, PFS2, and OS) for each treatment sequence, including median estimates and corresponding 95% confidence intervals. Missing demographic and baseline data were typical of this type of study and were not expected to substantially influence results. Statistical comparisons of efficacy outcomes were not performed; therefore, missing data imputation methods were not applied (no sensitivity analyses), and subgroups were constructed based on medical criteria (ie, treatment sequence received).

## 3 Results

### 3.1 Patients overall

Data from 727 patients in the PCR were eligible for inclusion, with seven treatment sequences analysed: abiraterone-docetaxel (ABI-DOCE; *n* = 178), abiraterone-enzalutamide (ABI-ENZA; *n* = 99), abiraterone–radium-223 (ABI-RAD; *n* = 27), docetaxel-abiraterone (DOCE-ABI; *n* = 191), docetaxel-cabazitaxel (DOCE-CABA; *n* = 74), docetaxel-enzalutamide (DOCE-ENZA; *n* = 116), and enzalutamide-docetaxel (ENZA-DOCE; *n* = 42). The study flow is shown in Supplementary Figure 2.

### 3.2 Patients who received first-line abiraterone

#### 3.2.1 Baseline characteristics

The median age of patients in the group first treated with abiraterone was 74.0 yr in both the ABI-DOCE and the ABI-RAD sequences and 78.0 yr in the ABI-ENZA sequence. Across sequences, the proportion of patients ≥75 yr of age was 44.9–66.7% (Table 1). The proportion of patients with visceral metastases ranged from 4.3% (ABI-RAD) to 12.3% (ABI-ENZA), that with M1 disease at initial diagnosis ranged from 24.5% (ABI-ENZA) to 43.4% (ABI-DOCE), and that with five or more bone metastases ranged from 35.7% (ABI-DOCE) to 50.0% (ABI-RAD; Table 1). In the ABI-ENZA, ABI-DOCE, and ABI-RAD sequences, respectively, 47.7%, 48.7%, and 59.3% of patients had Gleason scores ≥8 (Table 1), and 9.7%, 6.5%, and 3.7% of patients had ECOG performance status ≥2 (Table 2).

**Table 1:** Demographics and disease characteristics

	Treatment sequence						
	ABI-DOCE (*n* = 178)	ABI-ENZA (*n* = 99)	ABI-RAD (*n* = 27)	DOCE-ABI (*n* = 191)	DOCE-CABA (*n* = 74)	DOCE-ENZA (*n* = 116)	ENZA-DOCE (*n* = 42)
Age (yr)							
Median	74.0	78.0	74.0	69.0	68.5	71.0	70.5
Range	(50–92)	(48–94)	(61–92)	(46–87)	(53–80)	(47–85)	(54–88)
Age group (yr), *n* (%)							
<65	29 (16.3)	10 (10.1)	4 (14.8)	57 (29.8)	25 (33.8)	26 (22.4)	11 (26.2)
65–74	69 (38.8)	23 (23.2)	10 (37.1)	96 (50.3)	35 (47.3)	49 (42.3)	18 (42.8)
≥75	80 (44.9)	66 (66.7)	13 (48.1)	38 (19.9)	14 (18.9)	41 (35.3)	13 (31.0)
Gleason score at diagnosis, *n* (%)							
*N*	158	86	27	180	72	114	40
≤6	18 (11.4)	16 (18.6)	3 (11.1)	26 (14.4)	5 (6.9)	7 (6.1)	3 (7.5)
7	63 (39.9)	29 (33.7)	8 (29.6)	52 (28.9)	23 (31.9)	39 (34.2)	13 (32.5)
8–10	77 (48.7)	41 (47.7)	16 (59.3)	102 (56.7)	44 (61.1)	68 (59.6)	24 (60.0)
M stage at initial diagnosis, *n* (%)							
*N*	173	98	27	191	71	113	41
Mx	30 (17.3)	17 (17.3)	6 (22.2)	34 (17.8)	12 (16.9)	16 (14.2)	10 (24.4)
M0	68 (39.3)	57 (58.2)	11 (40.7)	68 (35.6)	24 (33.8)	36 (31.9)	16 (39.0)
M1, M1a, M1b, M1c	75 (43.4)	24 (24.5)	10 (37.1)	89 (46.6)	35 (49.3)	61 (53.9)	15 (36.6)
Time from initial diagnosis of prostate cancer to first metastatic diagnosis (yr)							
*N*	175	95	26	181	71	113	42
Median	0.8	3.4	2.9	0.3	0.1	0.1	0.9
Range	(0–24)	(0–21)	(0–16)	(0–17)	(0–11)	(0–15)	(0–19)
Presence of bone metastases, *n* (%)							
*N*	126	77	22	141	56	96	36
Any	83 (65.8)	55 (71.5)	17 (77.2)	82 (58.2)	38 (67.8)	57 (59.4)	19 (52.8)
≥5	45 (35.7)	29 (37.7)	11 (50.0)	52 (36.9)	25 (44.6)	34 (35.4)	9 (25.0)
Present, number unknown	28 (22.2)	14 (18.2)	5 (22.7)	41 (29.1)	16 (28.6)	26 (27.1)	9 (25.0)
Visceral metastases, *n* (%)							
*N*	145	81	23	169	66	95	34
Liver only	4 (2.8)	4 (4.9)	–	13 (7.7)	7 (10.6)	8 (8.4)	2 (5.9)
Lung only	9 (6.2)	5 (6.2)	1 (4.3)	11 (6.5)	5 (7.6)	3 (3.2)	1 (2.9)
Liver and lung	1 (0.7)	1 (1.2)	–	3 (1.8)	2 (3.0)	2 (2.1)	–

ABI = abiraterone acetate plus prednisone/prednisolone; CABA = cabazitaxel; DOCE = docetaxel; ENZA = enzalutamide; RAD = radium-223.

**Table 2:** Disease and health-related quality of life characteristics at baseline, start of second-line therapy, and change from baseline

	Treatment sequence						
	ABI-DOCE (*n* = 178)	ABI-ENZA (*n* = 99)	ABI-RAD (*n* = 27)	DOCE-ABI (*n* = 191)	DOCE-CABA (*n* = 74)	DOCE-ENZA (*n* = 116)	ENZA-DOCE (*n* = 42)
**ECOG performance status, *n* (%)**							
At baseline							
*N*	170	93	27	182	67	98	34
0	88 (51.8)	49 (52.7)	16 (59.3)	68 (37.4)	30 (44.8)	50 (51.0)	21 (61.8)
1	71 (41.8)	35 (37.6)	10 (37.0)	103 (56.6)	28 (41.8)	43 (43.9)	13 (38.2)
≥2	11 (6.5)	9 (9.7)	1 (3.7)	11 (6.0)	9 (13.4)	5 (5.1)	0
At start of second-line therapy							
*N*	151	87	23	169	64	87	24
0	38 (25.2)	30 (34.5)	6 (26.1)	36 (21.3)	13 (20.3)	23 (26.4)	6 (25.0)
1	88 (58.3)	39 (44.8)	11 (47.8)	113 (66.9)	29 (45.3)	39 (44.8)	13 (54.2)
≥2	25 (16.6)	18 (20.6)	6 (26.1)	20 (11.8)	22 (34.4)	25 (28.7)	5 (20.8)
Change from baseline							
*N*	146	83	23	167	61	83	22
Improved	12 (8.2)	5 (6.0)	1 (4.3)	14 (8.4)	1 (1.6)	4 (4.8)	1 (4.5)
No change	66 (45.2)	48 (57.8)	9 (39.1)	110 (65.9)	23 (37.7)	38 (45.8)	8 (36.4)
Worsened	68 (46.6)	30 (36.1)	13 (56.5)	43 (25.7)	37 (60.7)	41 (49.4)	13 (59.1)

**Prostate-specific antigen (ng/ml)**							
At baseline							
*N*	173	96	27	189	70	115	41
Median (range)	41.3 (1.5–5000.0)	33.8 (0.1–829.0)	21.9 (1.9–173.0)	42.4 (0.9–2108.0)	46.2 (1.0–2559.6)	34.9 (0.4–1900.0)	32.0 (2.5–1452.6)
At start of second-line therapy							
*N*	157	87	24	175	67	110	39
Median (range)	86.7 (0.2–5000.0)	52.5 (0.5–7000.0)	108.2 (6.2–853.0)	68.9 (0.1–2197.0)	81.0 (0.0–2887.5)	46.5 (0.4–3088.0)	59.5 (0.2–545.0)
Change from baseline							
*N*	149	82	24	165	61	109	39
Median (range)	33.2 (−149.1 to 1715.1)	15.1 (−653.4 to 6846.8)	49.8 (−77.0 to 595.0)	8.5 (−1874.9 to 1612.5)	1.1 (−842.8 to 971.0)	7.7 (−1864.6 to 2018.0)	10.0 (−907.6 to 332.0)

**Lactate dehydrogenase (U/l)**							
At baseline							
*N*	76	44	11	87	34	35	7
Median (range)	316.5 (134–1200)	209.0 (3–680)	301.0 (164–1207)	288.0 (5–1096)	328.0 (4–3232)	302.0 (177–815)	236.0 (183–371)
At start of second-line therapy							
*N*	78	44	10	62	35	40	10
Median (range)	323.5 (4–1947)	258.0 (156–1323)	342.0 (160–994)	287.5 (5–1346)	413.0 (3–6251)	287.0 (158–3460)	268.0 (179.0–452.0)
Change from baseline							
*N*	49	26	10	37	25	21	5
Median (range)	47.0 (−191 to 1332)	22.0 (−417 to 134)	16.0 (−461.0 to 551)	24.0 (−397 to 355)	134.0 (−2787 to 5557)	37.0 (−300 to 356)	−22.0 (−90.0 to 36.0)

**Haemoglobin (g/dl)**							
At baseline							
*N*	155	83	24	175	67	106	38
Median (range)	13.0 (7.5–15.9)	13.2 (9.1–16.9)	13.4 (9.1–15.9)	13.0 (7.4–16.1)	12.9 (7.3–16.3)	13.1 (7.4–15.5)	13.1 (9.8–15.8)
At start of second-line therapy							
*N*	152	74	24	165	63	105	38
Median (range)	12.0 (6.3–15.6)	12.2 (7.7–17.6)	12.4 (9.9–15.0)	12.0 (7.9–16.3)	11.8 (7.8–15.0)	12.0 (6.8–15.5)	12.2 (7.7–15.3)
Change from baseline							
*N*	137	64	23	148	60	96	35
Median (range)	−0.8 (−5.1 to 5.0)	−0.5 (−4.5 to 3.7)	−0.7 (−2.9 to 1.5)	−0.7 (−4.4 to 5.5)	−0.7 (−4.7 to 3.6)	−0.6 (−5.4 to 3.5)	−0.6 (−3.6 to 2.5)

**Alkaline phosphatase (U/l)**							
At baseline							
*N*	136	73	25	128	56	90	35
Median (range)	117.5 (1–1520)	93.0 (2–2337)	122.0 (42–1687)	104.5 (3–1850)	129.0 (1–3092)	119.5 (6–1429)	88.0 (22–5785)
At start of second-line therapy							
*N*	133	64	21	115	48	96	35
Median (range)	173.0 (44–2016)	104.0 (32–1659)	178.0 (37–1587)	135.0 (2–3728)	155.0 (1–1985)	130.5 (11–3510)	101.0 (17–3756)
Change from baseline							
*N*	113	52	21	84	39	81	34
Median (range)	27.0 (−414 to 1465)	10.7 (−159 to 1546)	30.0 (−941 to 1000)	3.0 (−1130 to 1878)	0.6 (−621 to 1774)	2.0 (−1548 to 3440)	0.0 (−2029 to 1253)

**EQ-5D-5L, pain/discomfort dimension, *n* (%)**							
At baseline							
*N*	126	72	22	144	58	80	35
1: No pain	46 (36.5)	32 (44.4)	5 (22.7)	35 (24.3)	14 (24.1)	21 (26.3)	9 (25.7)
2: Slight pain	48 (38.1)	24 (33.3)	12 (54.5)	50 (34.7)	23 (39.7)	33 (41.3)	17 (48.6)
3: Moderate pain	24 (19.0)	12 (16.7)	4 (18.2)	44 (30.6)	15 (25.9)	21 (26.3)	9 (25.7)
4: Severe pain	8 (6.3)	4 (5.6)	1 (4.5)	15 (10.4)	6 (10.3)	3 (3.8)	0
5: Extreme pain	0	0	0	0	0	2 (2.5)	0
At start of second-line therapy							
*N*	80	43	11	69	38	60	15
1: No pain or discomfort	22 (27.5)	12 (27.9)	0	13 (18.8)	5 (13.2)	15 (25.0)	3 (20.0)
2: Slight pain or discomfort	22 (27.5)	12 (27.9)	6 (54.5)	28 (40.6)	14 (36.8)	22 (36.7)	3 (20.0)
3: Moderate pain or discomfort	27 (33.8)	13 (30.2)	5 (45.5)	21 (30.4)	13 (34.2)	21 (35.0)	7 (46.7)
4: Severe pain or discomfort	9 (11.3)	5 (11.6)	0	6 (8.7)	4 (10.5)	2 (3.3)	1 (6.7)
5: Extreme pain or discomfort	0	1 (2.3)	0	1 (1.4)	2 (5.3)	0	1 (6.7)
Change from baseline							
*N*	69	36	8	57	33	38	9
Improved	16 (23.2)	2 (5.6)	0	17 (29.8)	6 (18.2)	11 (28.9)	2 (22.2)
No change	27 (39.1)	20 (55.6)	4 (50.0)	25 (43.9)	13 (39.4)	19 (50.0)	3 (33.3)
Worsened	26 (37.7)	14 (38.9)	4 (50.0)	15 (26.3)	14 (42.4)	8 (21.1)	4 (44.4)

**EQ-5D-5L, VAS**							
At baseline							
*N*	124	70	19	144	56	80	34
Median (range)	75.0 (20–100)	70.0 (25–95)	75.0 (30–100)	67.5 (0–100)	70.0 (30–100)	75.0 (10–98)	75.0 (40–100)
At start of second-line therapy							
*N*	78	43	10	67	38	60	15
Median (range)	70.0 (0–99)	60.0 (18–90)	72.5 (50–90)	65.0 (10–95)	60.0 (20–95)	70.0 (15–99)	55.0 (20–90)
Change from baseline							
*N*	68	38	7	56	33	39	10
Median (range)	0.0 (−65 to 40)	−10.0 (−52 to 15)	−5.0 (−10 to 10)	0.0 (−50 to 38)	−5.0 (−50 to 30)	−10.0 (−60 to 30)	−15.0 (−70 to 15)

ABI = abiraterone acetate plus prednisone/prednisolone; CABA = cabazitaxel; DOCE = docetaxel; ECOG = Eastern Cooperative Oncology Group; ENZA = enzalutamide; EQ-5D-5L = EuroQol 5-dimension 5-level questionnaire; RAD = radium-223; VAS = visual analogue scale.

#### 3.2.2 Reasons for switching treatment

Disease progression (assessed by PSA and/or radiological and/or clinical methods) was the most frequently cited reason for initiating and stopping first-line abiraterone; toxicity was cited as the second most common reason for stopping therapy early (Supplementary Table 1).

#### 3.2.3 Efficacy outcomes

**Fig. 1:**
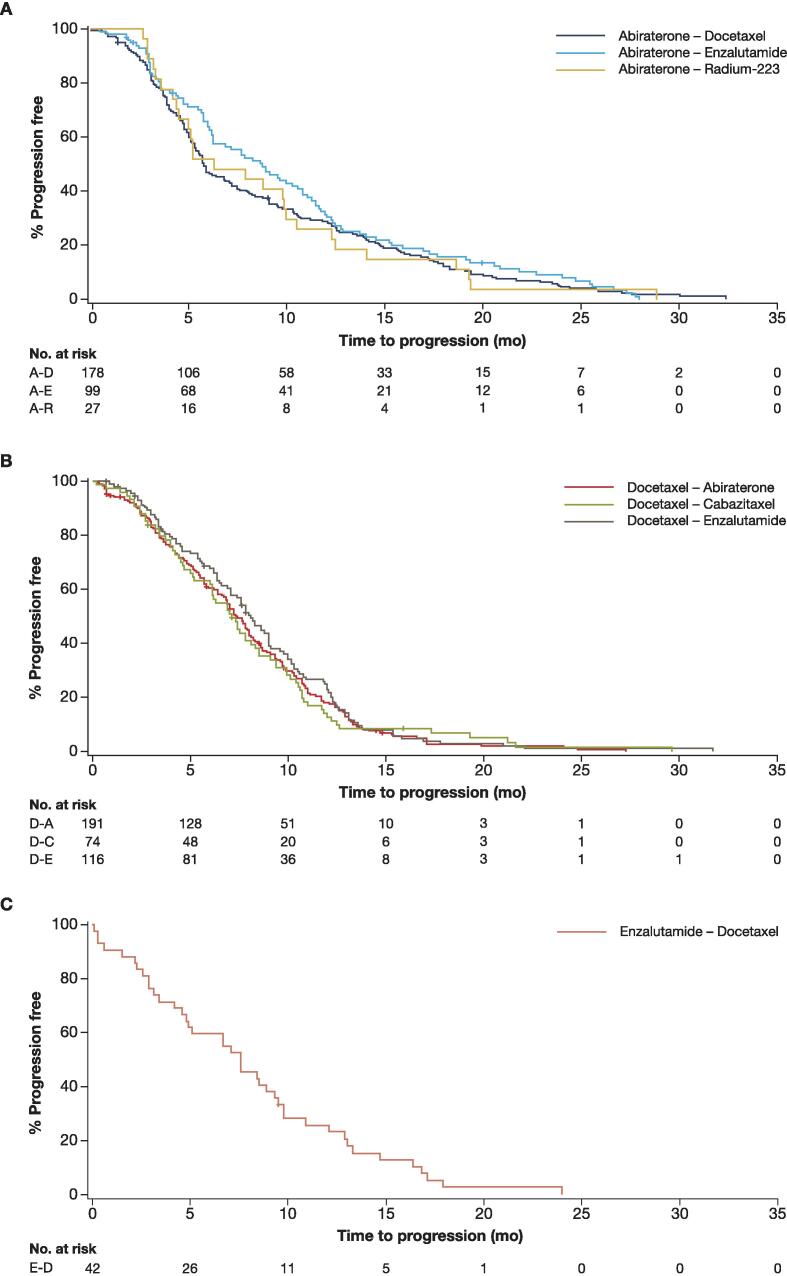
Time to progression. A-D = abiraterone-docetaxel; A-E = abiraterone-enzalutamide; A-R = abiraterone–radium-223.

**Fig. 2:**
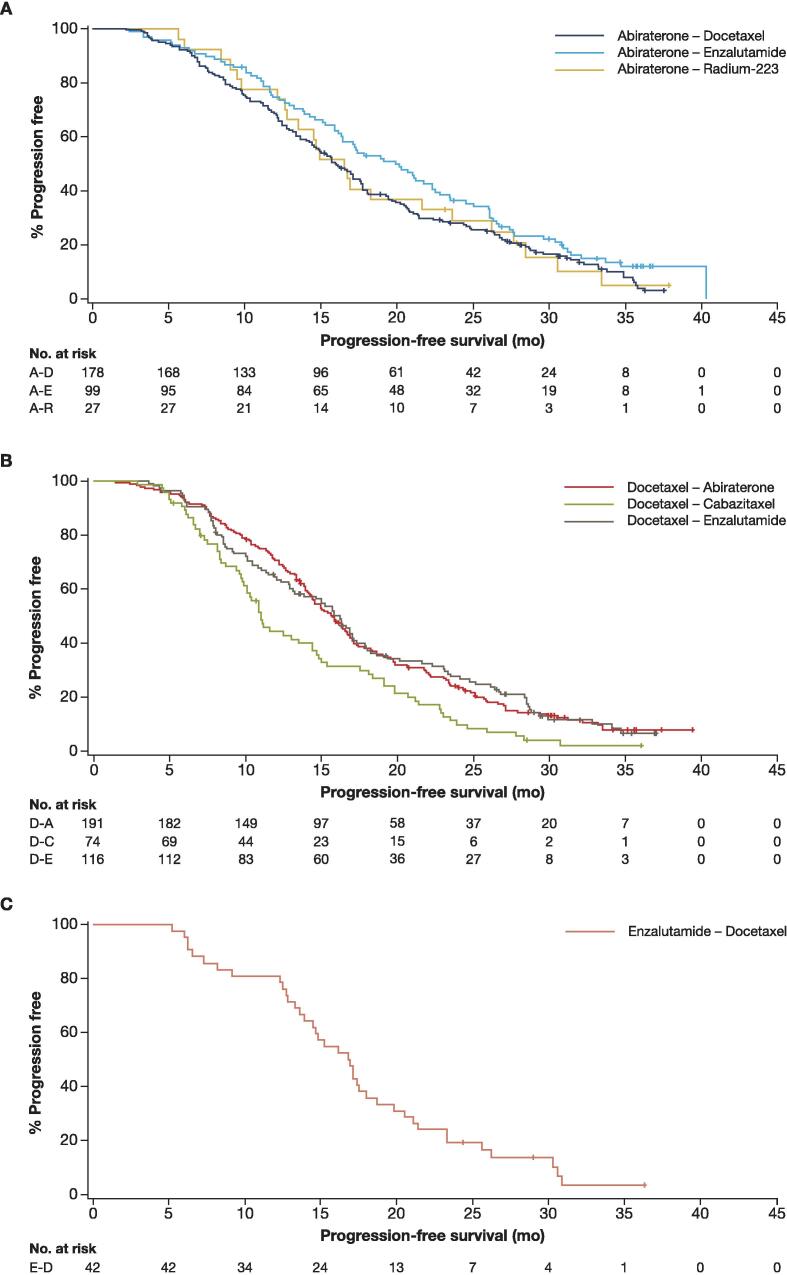
Progression-free survival 2. A-D = abiraterone-docetaxel; A-E = abiraterone-enzalutamide; A-R = abiraterone–radium-223.

**Fig. 3:**
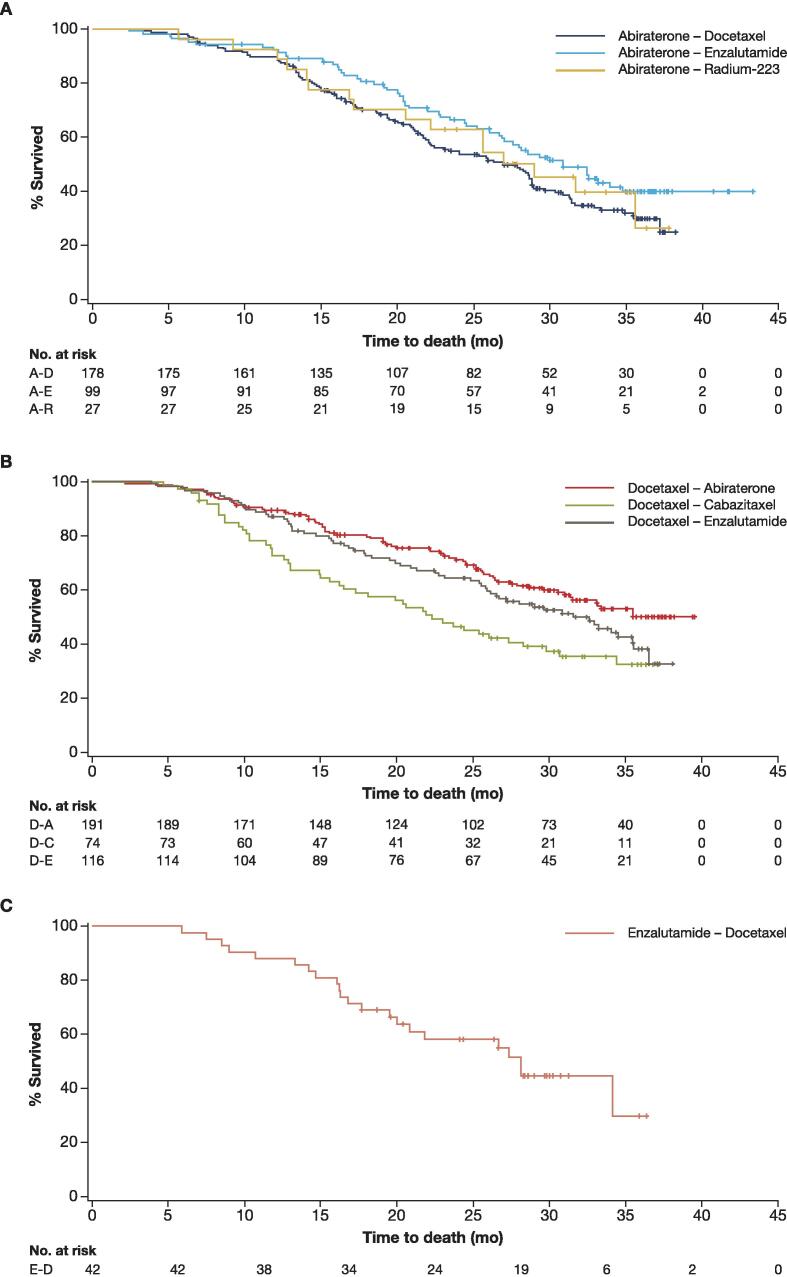
Overall survival. A-D = abiraterone-docetaxel; A-E = abiraterone-enzalutamide; A-R = abiraterone–radium-223.

The median TTP, PFS2, and OS (Kaplan-Meier estimates) ranged from 5.8, 15.9, and 27.0 mo, respectively, in the ABI-DOCE sequence to 8.7, 20.1, and 30.8 mo, respectively, in the ABI-ENZA sequence (Fig. 1, Fig. 2, Fig. 3 and Table 3). The median PSA, lactate dehydrogenase, and alkaline phosphatase increased from baseline to the start of second-line therapy in all sequences in the abiraterone group (Table 2).

**Table 3:** Efficacy outcomes

Treatment sequence	ABI-DOCE (*n* = 178)	ABI-ENZA (*n* = 99)	ABI-RAD (*n* = 27)	DOCE-ABI (*n* = 191)	DOCE-CABA (*n* = 74)	DOCE-ENZA (*n* = 116)	ENZA-DOCE (*n* = 42)
Time to progression (mo)^a^, median (95% CI)	5.8 (5.3–7.2)	8.7 (6.2–10.8)	6.2 (4.3–9.9)	7.4 (6.7–8.0)	7.1 (6.0–8.3)	8.1 (7.1–9.0)	7.6 (4.8–9.3)
Progression-free survival 2 (mo)^b^, median (95% CI)	15.9 (14.4–17.6)	20.1 (16.3–22.5)	16.5 (12.6–23.6)	15.7 (14.3–16.8)	11.0 (9.9–14.4)	16.2 (13.1–17.1)	16.9 (13.9–18.0)
Overall survival (mo)^c^, median (95% CI)	27.0 (22.1–28.9)	30.8 (26.7–NE)	29.0 (17.1–NE)	NE (31.4–NE)	22.3 (16.2–29.8)	31.5 (25.9–35.5)	28.1 (20.0–NE)

ABI = abiraterone acetate plus prednisone/prednisolone; CABA = cabazitaxel; CI = confidence interval; DOCE = docetaxel; ENZA = enzalutamide; mCRPC = metastatic castration-resistant prostate cancer; NE = not estimable; RAD = radium-223.

a Measured from the start date of the first mCRPC treatment to the start date of the second treatment. Time to progression was censored after the start of the second treatment. Patients with no progression at the end of registry are censored.

b Measured from the start of first treatment to progression/death after the start of second treatment. Patients with no progression at the end of registry are censored.

c Measured from the start date of mCRPC treatment to the date of death (irrespective of cause); survival time of living patients was censored at the last date a patient was known to be alive (for those withdrawn from the study) or the end-of-registry date.

#### 3.2.4 Change from baseline to start of second-line therapy in disease and health-related quality of life characteristics

The proportion of patients with no change or improvement in ECOG performance status was 63.8% in the ABI-ENZA sequence, 53.4% in the ABI-DOCE sequence, and 43.4% in the ABI-RAD sequence (Table 2).

The proportion of patients with no change or improvement in EQ-5D-5L pain/discomfort dimension scores ranged from 62.3% in the ABI-DOCE sequence to 50.0% in the ABI-RAD sequence (Table 2).

### 3.3 Patients who received first-line docetaxel

#### 3.3.1 Baseline characteristics

In the first-line docetaxel sequences, the median patient age ranged from 68.5 yr in the DOCE-CABA sequence to 71.0 yr in the DOCE-ENZA sequence, and 18.9–35.3% of patients were aged ≥75 yr across sequences (Table 1). The proportion of patients with visceral metastases ranged from 13.7% (DOCE-ENZA) to 21.2% (DOCE-CABA), and with M1 disease at initial diagnosis ranged from 46.6% (DOCE-ABI) to 53.9% (DOCE-ENZA). The proportions of patients with five or more bone metastases were 35.4%, 36.9%, and 44.6% in the DOCE-ENZA, DOCE-ABI, and DOCE-CABA sequences, respectively. The proportions of patients with ECOG performance status ≥2 were 5.1%, 6.0%, and 13.4% (Table 2) and those with Gleason score ≥8 were 59.6%, 56.7%, and 61.1% (Table 1) in the respective sequences.

#### 3.3.2 Reasons for switching treatment

Completion of therapy was the main reason for stopping first-line treatment, followed by disease progression; the main reason for initiating second-line therapy was disease progression (Supplementary Table 1).

#### 3.3.3 Efficacy outcomes

The median TTP, PFS2, and OS (Kaplan-Meier estimates) ranged, respectively, from 7.1, 11.0, and 22.3 mo in the DOCE-CABA sequence to 8.1, 16.2, and 31.5 mo in the DOCE-ENZA sequence (Fig. 1, Fig. 2, Fig. 3 and Table 3).

#### 3.3.4 Change from baseline to start of second-line therapy in disease and health-related quality of life characteristics

The proportion of patients with no change or improvement in ECOG performance status from baseline to the start of second-line therapy ranged from 74.3% in the DOCE-ABI sequence to 39.3% in the DOCE-CABA sequence (Table 2).

The proportion of patients with no change or improvement in EQ-5D-5L pain/discomfort dimension scores from baseline to the start of second-line therapy was 78.9% in the DOCE-ENZA sequence, 73.7% in the DOCE-ABI sequence, and 57.6% in the DOCE-CABA sequence (Table 2).

### 3.4 Patients who received first-line enzalutamide

#### 3.4.1 Baseline characteristics

The median age of patients at baseline was 70.5 yr, with 31.0% of patients aged ≥75 yr. The proportion of patients with five or more bone metastases was 25.0% and that with visceral metastases was 8.8% (Table 1). No patients in this group had ECOG performance status ≥2 (Table 2).

#### 3.4.2 Reasons for switching treatment

Disease progression was the most frequently cited reason for stopping first-line enzalutamide (Supplementary Table 1). All patients who initiated second-line docetaxel after enzalutamide did so because of disease progression (Supplementary Table 1).

#### 3.4.3 Efficacy outcomes

According to the Kaplan-Meier analysis, the median TTP was 7.6 mo (Fig. 1), median PFS2 was 16.9 mo (Fig. 2), and median OS was 28.1 mo (Fig. 3 and Table 3).

#### 3.4.4 Change from baseline to start of second-line therapy in disease and health-related quality of life characteristics

There was no change or improvement in ECOG performance status from baseline to the start of second-line therapy in 40.9% of patients (Table 2). In the EQ-5D-5L pain/discomfort dimension, 55.5% of patients had no change or improvement from baseline to the start of second-line therapy.

## 4 Discussion

The aim of this analysis was to report the use of different treatment sequences in real-world clinical practice. This report evaluates the characteristics and outcomes of the largest international cohort of patients receiving the most frequently prescribed first- and second-line mCRPC treatment in a real-world setting and presents information from patients in 16 countries.

There was a high degree of variation in demographics and disease characteristics between the sequence groups. While it is interesting to note this variation, the nonrandomised nature of this study and variation in availability of treatments in different countries mean that formal comparisons between treatment sequences cannot be made. Overall, abiraterone appeared to be prescribed more commonly as a first-line treatment to older patients and to those with lower Gleason scores. In general, patients with M1 disease at initial diagnosis with visceral metastasis were more commonly treated with first-line docetaxel than other first-line treatments. Patients with five or more bone metastases and those with visceral metastases appeared less likely to receive first-line enzalutamide. Baseline ECOG performance status scores were lowest in the first-line enzalutamide group. Thus, baseline patient characteristics could play a role in the selection of first-line treatment for mCRPC in the real-world setting. This was suggested in the ABI-RAD sequence, which appeared to be more commonly given to patients with five or more bone metastases, and only one patient with visceral metastases was given this treatment sequence; this is in accordance with the supporting indication for radium-233 in mCRPC [11]. There appeared to be a preference for first-line treatment with docetaxel in patients with visceral metastases and in patients of a younger age, likely because younger patients would be expected to tolerate docetaxel better than older patients [12]. In this context, it is rather surprising that the highest proportion of patients with ECOG performance status ≥2 was in the DOC-CABA sequence, although this was possibly influenced by reimbursement of the treatment options in different countries.

Efficacy outcomes were strictly observational, and no statistical comparisons were made between the treatment groups, as this was not a randomised study. We observed variation in TTP, PFS2, and OS within the first-line abiraterone group, with the longest duration of these seen when enzalutamide was given as second-line treatment. In patients who received first-line docetaxel, the TTP was similar between sequences; PFS2 and OS appeared shorter in patients who received second-line cabazitaxel, which could be influenced by the slightly higher proportion of patients with visceral metastases or ECOG performance status ≥2 in this sequence. Treatment groups were small, nonrandomised, and not stratified by patient characteristics; therefore, any differences observed in efficacy outcomes should be interpreted with caution. The efficacy data observed aid the understanding of mCRPC treatments in the real-world setting but cannot indicate an optimal treatment sequence in this patient population.

These results from the prospective analysis of data from the PCR can be placed in context of a small number of largely retrospective studies specifically investigating treatment sequencing outcomes for patients with mCRPC; however, methodological limitations restrict the interpretation of the findings from these studies. A recent open-label, phase II, crossover trial that was limited to a single country and included just 202 patients suggested a clinical benefit for abiraterone followed by enzalutamide relative to an ENZA-ABI sequence [13]. However, this benefit was limited to PSA-based endpoints, and no significant difference in OS was found between treatment sequences [14]. Outcomes with enzalutamide following abiraterone have also been compared between 115 docetaxel-experienced and docetaxel-naive patients in a Canadian study [15]. This study reported that the antitumour activity of ABI-ENZA was limited in patients with mCRPC, notwithstanding prior docetaxel, suggesting that earlier treatment with docetaxel does not lead to efficacy benefits for the ABI-ENZA sequence.

### 4.1 Limitations

This was an observational study, with no randomisation to allow treatment/sequence comparisons. As treatment followed routine clinical practice, duration of exposure varied with treatment cycle, and although some visits were performed according to the study protocol, routine clinical visits also resulted in data collection. These visits may not have occurred at regular intervals for all patients. The staggered commercial availability of the treatments in participating countries and differences in reimbursement and access to high-cost medication present potential for a bias, for example, newer treatments such as abiraterone and enzalutamide may not have been available in some countries at the time of data collection. Additionally, differences in treatment practices and settings (eg, among physicians [oncologists or urologists] and also practitioners in different countries) and timing of enrolment may have influenced the findings. Furthermore, patients’ preference of treatment may have been considered by treating physicians; however, this information was not collected. It is not possible to compare the efficacy of the different drug combinations statistically because of differences in demographics and patient characteristics in the treatment groups.

## 5 Conclusions

This analysis of real-world baseline characteristics, treatment patterns, and effectiveness outcomes in patients with mCRPC provides data on real-life application of treatment sequencing for mCRPC. The differences we observed in baseline patient characteristics suggest that these play an evident role in the selection of first-line treatment for mCRPC in the real-world setting, but we acknowledge that other factors, such as treatment availability, have an impact. The efficacy data observed provide good insight into real-world treatments for mCRPC, but are limited and cannot guide optimal treatment sequencing in this patient population.

  ***Author contributions*:** Anders Bjartell had full access to all the data in the study and takes responsibility for the integrity of the data and the accuracy of the data analysis.

*Study concept and design*: Wapenaar.

*Acquisition of data*: Not applicable.

*Analysis and interpretation of data*: Bjartell, Costa, Kramer, Zurawski, Galli, Werbrouck, Ecke, Parikh, Bennamoun, Freire, Peer, Ljungberg, Cicin, Smith, Lukac, Wapenaar, Chowdhury.

*Drafting of the manuscript*: Bjartell, Costa, Kramer, Zurawski, Galli, Werbrouck, Ecke, Parikh, Bennamoun, Freire, Peer, Ljungberg, Cicin, Smith, Lukac, Wapenaar, Chowdhury.

*Critical revision of the manuscript for important intellectual content*: Bjartell, Costa, Kramer, Zurawski, Galli, Werbrouck, Ecke, Parikh, Bennamoun, Freire, Peer, Ljungberg, Cicin, Smith, Lukac, Wapenaar, Chowdhury.

*Statistical analysis*: Lukac and Wapenaar.

*Obtaining funding*: Not applicable.

*Administrative, technical, or material support*: Not applicable.

*Supervision*: Not applicable.

*Other*: Not applicable.

  ***Financial disclosures:*** Anders Bjartell certifies that all conflicts of interest, including specific financial interests and relationships and affiliations relevant to the subject matter or materials discussed in the manuscript (eg, employment/affiliation, grants or funding, consultancies, honoraria, stock ownership or options, expert testimony, royalties, or patents filed, received, or pending), are the following: Anders Bjartell: payment or honoraria for lectures, presentations, speakers bureaus, manuscript writing, or educational events—Astellas, Bayer, Ferring, Ipsen, Janssen, Sandoz, and Recordati; participation in advisory board—Bayer, Janssen, AstraZeneca, and Merck; consulting fees—Ferring, Janssen, and AstraZeneca; shareholder—Glactone Pharma, LIDDS, and WntResearch. Luis Costa: consulting or advisory role—Ipsen, Novartis, and Roche; travel, accommodation, and expenses—Merck, Sharp & Dohme, Roche. Gero Kramer: grants/research support—Bayer and Sanofi Genzyme; honoraria or consultation fee—Astellas, AstraZeneca, Bayer, BMS, Ipsen, Janssen, MSD, Novartis, Sanofi Genzyme, Takeda, and Ferring. Bogdan Zurawski: salaries—Janssen Cilag, AstraZeneca, BMS, MSD, Bayer, Amgen, Novartis, and Astellas. Luca Galli: advisory role—BMS, MSD, Merck, Janssen Oncology, Pfizer, and AstraZeneca; honoraria—seminar/talks to industry—BMS, MSD, Merck, Janssen Oncology, Pfizer, AstraZeneca, Ipsen, and Takeda. Patrick Werbrouck, Thorsten Ecke, Omi Parikh, Mostefa Bennamoun, and Camilo Garcia Freire: none. Avivit Peer: consulting fees—MSD, BMS, Pfizer, Janssen, Astellas, Eisai, Bayer, AstraZeneca, and Roche; payment or honoraria for lectures, presentations, speakers bureaus, manuscript writing, or educational events—MSD, BMS, Pfizer, Janssen, Astellas, Eisai, Bayer, AstraZeneca, Roche, and Medison Pharma; support for attending meetings and/or travel—BMS, Astellas, Pfizer, and MSD; stock or stock options—Itamar Medical Inc. Börje Ljungberg: company speaker honorarium—Novartis, Pfizer, Ipsen, and BMS; trial participation and department honorarium—Janssen, Astellas, and Medivation; advisory board—Janssen, Ipsen, and MSD. Irfan Cicin: advisory board member—AbbVie, AstraZeneca, Boehringer Ingelheim, Eli Lilly, F. Hoffmann-La Roche, Merck Sharp & Dohme, and Pfizer; personal fees for lectures—Eli Lilly, Merck Sharp & Dohme, Novartis, Pfizer, Quintiles, and Roche; funding for the institution to support trial conduct—Astellas, AstraZeneca, BMS, Boehringer Ingelheim, Eli Lilly, Merck Serono, Merck Sharp & Dohme, Parexel, Pfizer, Quintiles, and Taiho. Emma Smith: full-time employee of Janssen. Martin Lukac: employee of Parexel International Czech Republic s.r.o., on behalf of Janssen Pharmaceutica N.V., Beerse, Belgium. Robert Wapenaar: full-time employee of Janssen and stockholder of Johnson & Johnson. Simon Chowdhury: consulting fees—Telix, Remedy, and Huma; payment or honoraria for lectures, presentations, speakers’ bureaus, manuscript writing, or educational events—AstraZeneca, Novartis/AAA, Clovis, Janssen, Bayer, Pfizer, Beigene, and Astellas.

  ***Funding/Support and role of the sponsor*:** This study was funded by Janssen EMEA. Janssen EMEA contributed to the study design; collection, analysis, and interpretation of data; writing of the report; and decision to submit the paper for publication.

  ***Acknowledgments:*** Editorial assistance in the development of this manuscript was provided by Kate Bradford, PhD, of Parexel, funded by Janssen-Cilag.
