# Supplementary material for: Real-world Treatment Sequencing in Patients with Metastatic Castration-resistant Prostate Cancer: Results from the Prospective, International, Observational Prostate Cancer Registry
Source: Eur Urol Open Sci. 2022 Sep 17;45:12–22. doi: 10.1016/j.euros.2022.08.018 (PMC9637537; doi:10.1016/j.euros.2022.08.018)
Supplement: Supplementary data 1 [file mmc1.docx]

# Supplementary material

**Definitions and criteria used for calculating efficacy outcomes**

Time to progression was measured from the start date of the first metastatic castration-resistant prostate cancer (mCRPC) treatment to the start date of the second treatment.

Criteria for disease progression on treatment were met if: 1) there was evidence of radiographic progression per investigator assessment (guidelines from the Prostate Cancer Working Group 2 and Response Evaluation Criteria in Solid Tumors were recommended in the protocol), 2) there was evidence of clinical progression per investigator assessment, 3) first-line mCRPC treatment was stopped because of progression, or 4) second-line mCRPC treatment was started because of progression. Patients with no progression at end of registry were censored.

Progression-free survival 2 (PFS2) was calculated from the start date of the first treatment of the sequence to the date of progression or death after the start date of the second treatment of the sequence. If no event occurred, then censoring was at the start date of the third therapy or at the end-of-registry date, whichever came first.

Overall survival (OS) was measured from the start date of mCRPC treatment to the date of death (irrespective of cause). Survival time of living patients was censored at the last date a patient was known to be alive (for those withdrawn from the study) or the end of registry.

Supplementary Figure 1 – Study timeline


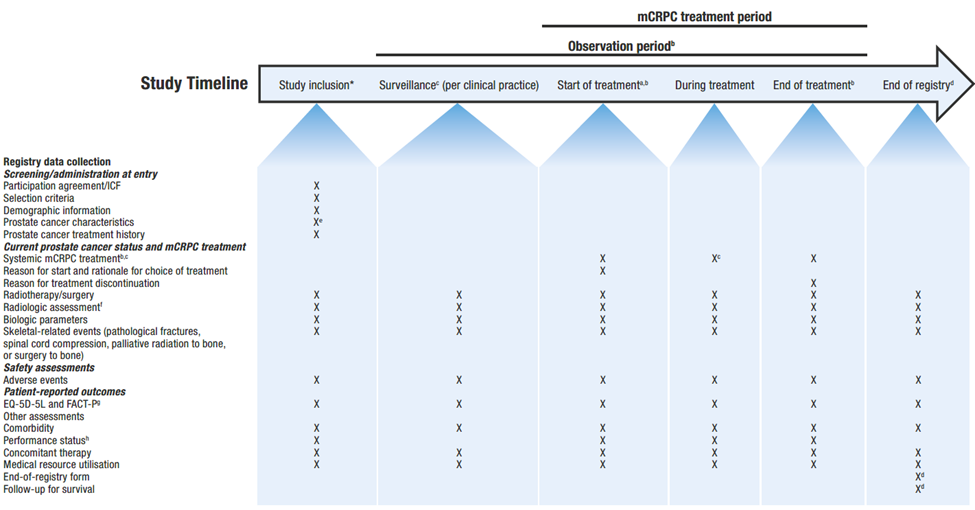


Note: Patients could enter multiple systemic mCRPC treatment periods and periods of surveillance during the registry.

ECOG = Eastern Cooperative Oncology Group; EQ-5D-5L = European Quality of Life-5 Dimensions, 5 Levels; FACT-P = Functional Assessment of Cancer Therapy-Prostate; ICF = International Classification of Functioning, Disability and Health; TNM = tumor, node, metastasis; mCRPC = metastatic castration-resistant prostate cancer.

^a^For patients entering the registry, the data from the baseline data collection and start of data collection may be the same; at enrolment, initiation of a new systemic mCRPC treatment was considered at ±30 days from a patient’s baseline data collection.

^b^Data were collected at the points of initiation and termination of each new systemic mCRPC treatment during the observational period. Recorded information included the type of treatment, start and stop dates, dose, and frequency of administration. The maximum duration of follow-up for individual patients in the observational period of the registry was 3 years, regardless of when they enrolled.

^c^For patients undergoing long-term (>3 months) systemic mCRPC treatment or in surveillance, data were collected at suggested intervals of 3 months.

^d^The end of registry was the last data collection time point for an individual patient, a maximum of 3 years after their enrolment. The close of registry will be approximately 5.5 years after the first patient was enrolled. Survival data will be collected for all patients 3 years after their enrolment or at the close of the registry, whichever occurs first, except for those patients who withdraw their consent prior to completing the registry.

^e^Including dates of initial diagnosis, first metastatic diagnosis and castration resistance, TNM stage and Gleason score at diagnosis, and most recent Gleason score.

^f^Prostate Cancer Working Group 2 and Response Evaluation Criteria In Solid Tumors are recommended guidelines for radiologic assessment.

^g^Where permitted per local regulations.

^h^Performance status was collected using the ECOG scale.

Supplementary Figure 2 – Overview of patients and study flow


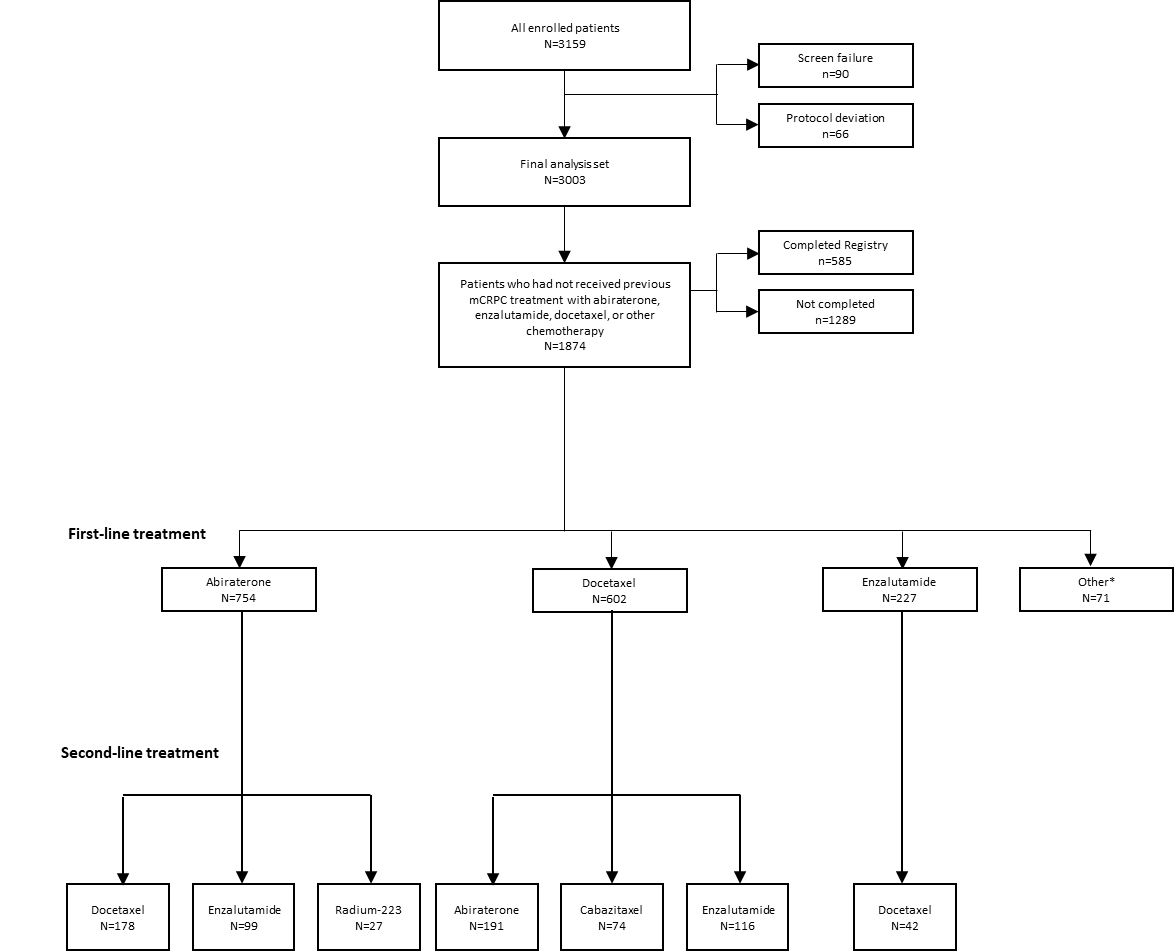


Only data from patients who received abiraterone acetate plus prednisone, docetaxel, or enzalutamide as first line in their treatment sequence were analysed.

*Bevacizumab, cabazitaxel, carboplatin, cyclophosphamide, epirubicin, estramustine, etoposide, everolimus, fluorouracil, gemcitabine, methotrexate, mitoxantrone, oxaliplatin, paclitaxel, pemetrexed, radium-223, vinblastine, vinorelbine or trial medication.

Supplementary Table 1 – Reasons for starting and stopping systemic mCRPC treatment

|  | Treatment sequence | | | | | | |
| --- | --- | --- | --- | --- | --- | --- | --- |
|  | ABI-DOCE (*n* = 178) | ABI-ENZA (*n* = 99) | ABI-RAD (*n* = 27) | DOCE-ABI (*n* = 191) | DOCE-CABA (*n* = 74) | DOCE-ENZA (*n* = 116) | ENZA-DOCE (*n* = 42) |
| *First-line treatment* | | | | | | | |
| Reason for starting treatment, *n* (%) | | | | | | | |
| Disease progression^a^ | 178 (100.0) | 98 (99.0) | 27 (100.0) | 188 (98.4) | 72 (97.3) | 115 (99.1) | 42 (100.0) |
| PSA | 151 (84.8) | 87 (87.9) | 22 (81.5) | 164 (85.9) | 57 (77.0) | 88 (75.9) | 34 (81.0) |
| Radiological | 116 (65.2) | 55 (55.6) | 17 (63.0) | 118 (61.8) | 57 (77.0) | 75 (64.7) | 25 (59.5) |
| Clinical | 38 (21.3) | 13 (13.1) | 5 (18.5) | 62 (32.5) | 26 (35.1) | 23 (19.8) | 8 (19.0) |
| Toxicity of previous therapy | – | 1 (1.0) | – | – | 1 (1.4) | – | – |
| Ongoing castration | – | – | – | 1 (0.5) | – | – | – |
| Ongoing palliative | – | – | – | – | – | 1 (0.9) | – |
| Other | – | – | – | 2 (1.0) | 1 (1.4) | – | – |
| Reason for stopping treatment, *n* (%) | | | | | | | |
| Completed therapy | 3 (1.7) | 1 (1.0) | 0 | 98 (51.3) | 38 (51.4) | 62 (53.4) | 0 |
| Toxicity^b^ | 8 (4.5) | 10 (10.1) | 1 (3.7) | 31 (16.2) | 6 (8.1) | 18 (15.5) | 1 (2.4) |
| Disease progression | 165 (92.7) | 82 (82.8) | 26 (96.3) | 52 (27.2) | 29 (39.2) | 29 (25.0) | 41 (97.6) |
| Death | 0 | 0 | 0 | 1 (0.5) | 0 | 0 | 0 |
| Other | 2 (1.1) | 6 (6.1) | 0 | 9 (4.7) | 1 (1.4) | 7 (6.0) | 0 |
| *Second-line treatment* | | | | | | | |
| Reason for starting treatment | | | | | | | |
| Disease progression^a^ | 169 (94.9) | 93 (93.9) | 26 (96.3) | 170 (89.0) | 70 (94.6) | 106 (91.4) | 42 (100.0) |
| PSA | 120 (67.4) | 67 (67.7) | 21 (77.8) | 141 (73.8) | 42 (56.8) | 80 (69.0) | 34 (81.0) |
| Radiological | 124 (69.7) | 49 (49.5) | 15 (55.6) | 109 (57.1) | 49 (66.2) | 50 (43.1) | 27 (64.3) |
| Clinical | 56 (31.5) | 30 (30.3) | 10 (37.0) | 56 (29.3) | 27 (36.5) | 23 (19.8) | 11 (26.2) |
| Toxicity of previous therapy | 6 (3.4) | 6 (6.1) | – | 12 (6.3) | 3 (4.1) | 8 (6.9) | – |
| Ongoing palliative | 1 (0.6) | – | 1 (3.7) | – | – | 1 (0.9) | – |
| Other | 2 (1.1) | – | – | 7 (3.7) | – | 1 (0.9) | – |
| Ongoing bone sparing | – | – | – | 2 (1.0) | 1 (1.4) | – | – |
| Reason for stopping treatment | | | | | | | |
| *N* | 163 | 73 | 23 | 150 | 71 | 91 | 36 |
| Completed therapy | 57 (35.0) | 1 (1.4) | 7 (30.4) | 3 (2.0) | 14 (19.7) | 4 (4.4) | 14 (38.9) |
| Toxicity^b^ | 30 (18.4) | 11 (15.1) | 1 (4.3) | 8 (5.3) | 12 (16.9) | 9 (9.9) | 5 (13.9) |
| Disease progression | 57 (35.0) | 44 (60.3) | 13 (56.5) | 112 (74.7) | 33 (46.5) | 66 (72.5) | 12 (33.3) |
| Death | 13 (8.0) | 10 (13.7) | 1 (4.3) | 8 (5.3) | 9 (12.7) | 5 (5.5) | 2 (5.6) |
| Other | 6 (3.7) | 7 (9.6) | 1 (4.3) | 19 (12.7) | 3 (4.2) | 7 (7.7) | 3 (8.3) |

ABI = abiraterone acetate plus prednisone/prednisolone; CABA = cabazitaxel; DOCE = docetaxel; ENZA = enzalutamide; PSA = prostate-specific antigen; RAD = radium-223.

^a^More than one type of disease progression could be cited; percentages for subcategories are calculated as *n*/*N* × 100.

^b^Toxicity data were collected only as a reason for stopping treatment without specifying type of toxicity.
